# Correction: Integrating psychosocial health into disaster risk management: Insights from COVID-19 in Durán, Ecuador

**DOI:** 10.1371/journal.pone.0353209

**Published:** 2026-07-06

**Authors:** Mercy J. Borbor-Cordova, Madison Searles, Heydi Roa-López, María del Pilar Cornejo-Rodriguez, Katty Castillo, Andrea Orellana-Manzano, Christina D. Campagna

Fig 3 was uploaded incorrectly. Please see the correct Fig 3 here.

**Fig 3:**
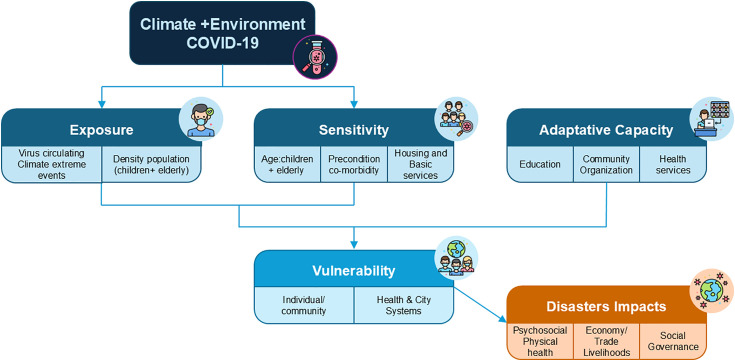
Diagram of the multi-hazard vulnerability framework, should consider multiple dimensions. Exposure refers to the degree to which populations, such as children and elders, are affected by climate-related disasters or viral outbreaks. Sensitivity is shaped by individual factors like age, disabilities, or pre-existing health conditions, urban factors such as access to basic services, housing conditions, and infrastructure. Adaptive capacity, or the ability to respond and recover, is influenced by levels of education, the presence of community organization, and the availability and quality of health and municipal services.
