# Supplementary material for: Integrating psychosocial health into disaster risk management: Insights from COVID-19 in Durán, Ecuador
Source: PLoS One. 2026 Mar 27;21(3):e0343239. doi: 10.1371/journal.pone.0343239 (PMC13029789; doi:10.1371/journal.pone.0343239)
Supplement: S1 File — (PDF) [file pone.0343239.s001.pdf]

SUPLEMENTARY MATERIAL:

S1_ a) **Durán survey questionnaire and instruments used to assess b) Perceived stress and c) Depression**

	a)	SURVEY APPLIED at Duran City for Disaster Crisis and Psychosocial impacts	
**#**	**Section**	**Description**	**Question**
	Participant Identification	Participant's Full Name	What is your full name? Please provide two first names and two last names. Please show your identity card.
		Nationality	What is the participant's nationality?
		Participant's date of birth	What is the participant's date of birth?
		Participant's Contact Number	What is the participant's contact number?
		Interview Date	
		Interviewer's name	N/A
		Participant's enrollment site	N/A
		Informed Consent	
		Confidentiality private	

#	Section	Valid Answer	Description	Question
	Participant Characteristics	>12 - 80	Participant's age, in years	How old is the participant?
		1 = Male<br>2 = Female	Participant's sex	Is the participant male or female?
		1 = Indigenous<br>2 = Afro-Ecuadorians<br>3 = Montubio or Mestizo<br>4 = White or Other<br>88 = Unknown<br>99 = No answer	Participant's ethnicity	What is the participant's ethnicity?

		Text	Participant's canton	In which canton does the participant live?
		Text	Participant's parish	In which parish does the participant live?
		Text	City of the participant's residence	In which city does the participant live?
		Text	Urbanization or locality of the participant's residence	In which urbanization or locality does the participant live?
	Travel history	0 = No<br>1 = Yes<br>88 = Unknown	Participant's travel history	Has the participant traveled in the last 4 weeks?
		Text	Participant's travel destination	If they have traveled, where have they traveled? If they are a migrant, where are they from?

	**Valid Answer**	**Description**	**Question**
Demographic data	1 = Married<br>2 = Widowed<br>3 = Divorced<br>4 = Separated<br>5 = Never married<br>6 = Unmarried, living with a partner<br>88 = Unknown<br>99 = No answer	What is the marital status of the representative ?	What is the marital status of the participant?
	1 = Primary education<br>2 = Secondary education<br>3 = Tertiary education<br>4 = Technical or university education<br>5 = Postgraduate studies<br>88 = Unknown	Highest level of education of the representative	What is the highest level of education attained by the participant?

	1 = Student<br>2 = Professional (teacher, nurse)<br>3 = Semi-professional (shop assistant)<br>4 = Skilled worker (construction)<br>5 = Unskilled worker (cleaner)<br>6 = Homemaker<br>7 = Unemployed<br>8 = Retired<br>9 = Other<br>88 = Unknown	Type of occupation	What is the participant's occupation?
Residence and basic services	1 = Private house (house or villa)<br>2 = Apartment in a house or building<br>3 = Room(s) in a boarding house<br>4 = Shack<br>5 = Ranch<br>6 = Hut<br>7 = Hovel<br>8 = Collective housing<br>9 = Other private dwelling<br>88 = Unknown<br>99 = No answer	Type of housing	What type of housing does the participant live in?
	1 = Connected to a public sewer system<br>2 = Connected to a septic tank<br>3 = Connected to a cesspit<br>4 = Direct discharge to the sea, river, lake, or stream<br>5 = Latrine<br>6 = Doesn't have one<br>88 = Unknown<br>99 = No answer	Sanitary conditions of the housing	What type of toilet facilities does your household have?
	1 = Collected by garbage truck<br>2 = Disposed of in a vacant lot or ravine<br>3 = Burned<br>4 = Buried<br>5 = Disposed of in a river, ditch, or canal<br>6 = In another way<br>88 = Unknown<br>99 = No answer	Waste disposal for the housing	How does your household dispose of garbage?

	1 = From public network<br>2 = From well<br>3 = From river, spring, ditch, or canal<br>4 = From water delivery truck<br>5 = Rainwater<br>6 = Other<br>88 = Unknown<br>99 = No answer	Water source for the housing	Where does the drinking water that arrives at the household come from?
	1 = They drink it as it comes into the home<br>2 = They boil it<br>3 = They add chlorine<br>4 = They filter it<br>5 = They buy purified water<br>88 = Unknown<br>99 = No answer	Water usage in the housing	Primarily, the water that household members drink:
	0 = No<br>1 = Yes<br>88 = Unknown<br>99 = No answer	Stagnant water near the house	Are there stagnant water deposits in your house or its surroundings ?
	0 = No<br>1 = Yes<br>88 = Unknown<br>99 = No answer	Mud near the house	Is there mud in your house or its surroundings ?
	0 = No<br>1 = Yes<br>88 = Unknown<br>99 = No answer	Mosquitoes near the house	Are mosquitoes frequently seen in your house or its surroundings ?

	Valid Answer	Description	Question
Sensitivity Morbidity	0 = No<br>1 = Yes<br>88 = Unknown	Chronic diseases	Do you have any chronic illnesses, such as diabetes, hypertension, high cholesterol, or elevated triglycerides?
	1 = Cardiovascular disease<br>2 = Cancer<br>3 = COPD<br>4 = Asthma<br>5 = Diabetes<br>88 = Unknown	Chronic diseases	Which non-communicable chronic illnesses do you have?

#	Sensitivity	Valid Answer	Description	Question
	COVID-19 Diagnostic	0 = No<br>1 = Yes<br>88 = Unknown	COVID-19 test	Have you received a laboratory test for COVID-19?
		0 = No<br>1 = Yes<br>88 = Unknown	COVID-19 test report	Do you have the laboratory report with the test?
		1 = Positive<br>2 = Negative<br>3 = Results not available<br>4 = Inconclusive<br>88 = Unknown	COVID-19 test result	What was the result of the test?
	Inmunization	0 = No<br>1 = Yes<br>88 = Unknown	COVID-19 Inmunization	Have you received the COVID-19 vaccine?
		0 = No<br>1 = Yes<br>88 = Unknown	Chronic diseases	Do you have any chronic illnesses, such as diabetes, hypertension, high cholesterol, or elevated triglycerides?

	Chronic Diseases	1 = Cardiovascular disease<br>2 = Cancer<br>3 = COPD<br>4 = Asthma<br>5 = Diabetes<br>88 = Unknown	Chronic diseases	Which non-communicable chronic illnesses do you have?
				Before this visit, has your doctor said that has any of the followiyoung illnesses:
	History of other possible infections or disease	0 = No<br>1 = Yes<br>88 = Unknown	History of HIV/AIDS, reported by the participant or their representative	HIV/AIDS?
		0 = No<br>1 = Yes<br>88 = Unknown	History of tuberculosis, reported by the participant or their representative	Tuberculosis?
		0 = No<br>1 = Yes<br>88 = Unknown	History of cancer, reported by the participant or their representative	Cancer?
		0 = No<br>1 = Yes<br>88 = Unknown	History of diabetes, reported by the participant or their representative	Diabetes?
		0 = No<br>1 = Yes<br>88 = Unknown	History of cardiovascular disease, reported by the participant or their representative	Cardiovascular disease?
		0 = No<br>1 = Yes<br>88 = Unknown	History of Chronic Obstructive Pulmonary Disease (COPD), reported by the participant or their representative	Chronic Obstructive Pulmonary Disease (COPD)?
		0 = No<br>1 = Yes<br>88 = Unknown	History of anemia, reported by the participant or their representative	Anemia?

	0 = No<br>1 = Yes<br>88 = Unknown	History of asthma, reported by the participant or their representative	Asthma?
	0 = No<br>1 = Yes<br>88 = Unknown	History of overweight, reported by the participant or their representative	Overweight?
	0 = No<br>1 = Yes<br>88 = Unknown	History of obesity, reported by the participant or their representative	Obesity?
	0 = No<br>1 = Yes<br>88 = Unknown	History of hypertension, reported by the participant or their representative	Hypertension?
	0 = No<br>1 = Yes<br>88 = Unknown	History of pneumonia, reported by the participant or their representative	Pneumonia?
Family history of the disease	0 = No<br>1 = Yes<br>88 = Unknown	Family history of COVID-19	In the last 15 days, has anyone in your household been diagnosed with COVID-19?
	0 = No<br>1 = Yes<br>88 = Unknown	Family history of COVID-19 symptoms	In the last 15 days, has anyone in your household shown symptoms (fever, nasal congestion, respiratory difficulty)?
			Have you use any of these recreational drugs?
Drug and Tabacco Use	0= No<br>1= Yes<br>99= No answer	Use of recreational drugs	Cannabis
	0= No<br>1= Yes<br>99= No answer	Use of recreational drugs	Heroine
	0= No<br>1= Yes<br>99= No answer	Use of recreational drugs	Cocaine
	0= No<br>1= Yes<br>99= No answer	Use of recreational drugs	Other

	0 = No<br>1 = Yes<br>3= never<br>99= No answer	Tabacco Use	Do you smoke or have smoked?
			If Yes, How often do you smoke?
	0= No<br>1= Yes<br>2= never<br>99= No answer	Frequency	1-3 times/week
	0= No<br>1= Yes<br>2= never<br>99= No answer	Frequency	2-3 times/month
	0= No<br>1= Yes<br>2= never<br>99= No answer	Frequency	Once a month or less

	**b) Self perceived Stress questionnaire**					
**#**	**Question**	**Never (0)**	**almost never (1)**	**sometimes (2)**	**fairly often (3)**	**very often (4)**
1	Have you felt nervous or stressed?					
2	How often have you felt confident about your ability to handle your personal problems?					
3	How often have you felt that things were going your way?					
4	Have you felt difficulty coping with all the things you have to do?					
5	Have you experienced feelings of anger or rage because things that happened were beyond your control?					
6	How often have you felt difficulties were piling up so high that you could not overcome them					

#	**c) Depression Questionnaire**	**Not at all (0)**	**Several days (1)**	**More than half the days (2)**	**Nearly every day (3)**
**1**	Little interest or pleasure in doing things				
**2**	Feeling down, depressed, or hopeless				
3	Trouble falling or staying asleep, or sleeping too much				
4	Feeling tired or having little energy				
5	Poor appetite or overeating				
6	Feeling bad about yourself - or that you are failure or have let yourself or your family down				
	Total	0	0	0	0
10	If you checked off any problems, how difficult have these problems made it for you to do your work, take care of things at home, or get along with other people?	Not difficult at all	_______________		
		Somewhat difficult	_______________		
		Very difficult	_______________		
		Extremely difficult	_______________		
	**Depression severity**	**Total Score**			
	Minimal depression	1-4			
	Mild depression	5-9			
	Moderate depression	10-14			
	Moderately severe depression	15-19			
	Severe depression	20-17			

b) Perceived Stress Scale (PSS – 6)

Adapted version of PSS-10

**Over the <u>last month</u>, how often have you been bothered by any of the following problems?** *(Use "✓" to indicate your answer)*	PSS-10* r i-x	PSS-10* Cronbach's α-x	PSS-6 r i-x	PSS-6* Cronbach's α-x
**1.** In the last month, how often have you felt nervous and stressed?	0.588	0.841	0.59	0.61
**2.** In the last month, how often have you felt confident about your ability to handle your personal problems?	0.525	0.847	0.50	0.65
**3.** In the last month, how often have you felt that things were going your way?	0.572	0.843	0.56	0.62
**4.** In the last month, how often you found that you could not cope with all the things that you had to do?	0.468	0.851	0.53	0.64
**5.** In the last month, how often have you been angered because of things that happened that were outside of your control?	0.547	0.845	0.71	0.54
**6.** In the last month, how often have you felt difficulties were piling up so high that you could not overcome them?	0.649	0.836	0.70	0.55

- Internal consistency of the PSS-10 on Ruisoto 2020

Scoring : Each item is scored on a five-point Likert scale from 0 to 4, except items 4 and 5 which are reverse scored.

Never Almost never Sometimes Fairly often Very Often

The levels of perceived stress were classified as follows:

**Score**	**Level of Perceived Stress**	**Internal consistency Cronbach's α**
0 - 8	Low	0.667
9 - 16	Moderate	
17 - 24	High	

This questionnaire was based on previous work applied in Ecuador.

Paz C, Abiuso T, Adana-Díaz L, Rodríguez-Lorenzana A, Jaramillo-Vivanco T, Ortiz-Prado E, et al. Psychological Distress in the Galapagos Islands During the COVID-19 Pandemic. Int J Public Health. 2022;67: 1604366

Ruisoto P, López-Guerra VM, Paladines MB, Vaca SL, Cacho R. Psychometric properties of the three versions of the Perceived Stress Scale in Ecuador. 2020;224: 113045. doi: 10.1016/j.physbeh.2020.113045

b) PSS - 6 Version in Spanish

**Durante el último mes, ¿qué tan seguido le han afectado cualquiera de los siguientes problemas?** *(Marque con una "✓" para indicar su respuesta)*
**1.** En el último mes, ¿con qué frecuencia se ha sentido nervioso y estresado?
**2.** En el último mes, ¿con qué frecuencia se ha sentido se ha sentido seguro de su capacidad para manejar sus problemas personales?
**3.** En el último mes, ¿con qué frecuencia has sentido que las cosas iban como tú querías?
**4.** En el último mes, ¿con qué frecuencia ha sentido que no podía hacer frente a todas las cosas que tenía que hacer?
**5.** En el último mes, ¿con qué frecuencia te has enfadado por cosas que han ocurrido y que estaban fuera de tu control?
**6.** En el último mes, ¿con qué frecuencia has sentido que las dificultades se acumulaban tanto que no podías superarlas?

c) Patient Health Questionnaire
PHQ-9 (Depression)

**Over the last 2 weeks, how often have you been bothered by any of the following problems?** *(Use "✓" to indicate your answer)*	PHO-9 $r_{i\text{-}x}$	PHQ-9 Cronbach's α-x
**1.** Little interest or pleasure in doing things	0.75	0,89
**2.** Feeling down, depressed, or hopeless	0.85	0.88
**3.** Trouble falling or staying asleep, or sleeping too much	0,69	0.90
**4.** Feeling tired or having little energy	0.78	0.89
**5.** Poor appetite or overeating	0.71	0.90
**6.** Feeling bad about yourself — or that you are a failure or have let yourself or your family down	0.81	0.89
**7.** Trouble concentrating on things, such as reading the newspaper or watching television	0.81	0.89
**8.** Moving or speaking so slowly that other people could have noticed? Or the opposite — being so fidgety or restless that you have been moving around a lot more than usual	0.79	0.89

Scoring : Each item is scored on a five-point Likert scale from 0 to 4, except items 4 and 5 which are reverse scored.

Not at all (0) Several days (1) More than half the days (2) Nearly every (3)

The levels of perceived stress were classified as follows:

**Score**	**Level of Depression**	**Internal consistency Cronbach's α**
0 - 9	Minimal to Mild	0.87
10 -14	Moderate	
15 - 19	Moderately Severe	
20 - 24	Severe	

Reference: Paz C, Mascialino G, Adana-Díaz L, Rodríguez-Lorenzana A, Simbaña-Rivera K, Gómez-Barreno L, et al. Behavioral and sociodemographic predictors of anxiety and depression in patients under epidemiological surveillance for COVID-19 in Ecuador. PLoS One. 2020;15: e0240008

Cuestionario sobre la salud del paciente (Depresión) (PHQ-9) Versión in Spanish

Durante las últimas 2 semanas, ¿qué tan seguido le han afectado cualquiera de los siguientes problemas?
(Marque con una "✓" para indicar su respuesta)

1. Poco interés o placer en hacer las cosas

2. Se ha sentido decaído(a), deprimido(a), o sin esperanzas

3. Dificultad para dormir o permanecer dormido(a), o ha dormido demasiado

4. Se ha sentido cansado(a) o con poca energía

5. Con poco apetito o ha comido en exceso

6. Se ha sentido mal con usted mismo(a) – o que es un fracaso o que ha quedado mal con usted mismo(a) o con su familia

7. Ha tenido dificultad para concentrarse en cosas tales como leer el periódico o ver televisión

8. ¿Se ha estado moviendo o hablando tan lento que otras personas podrían notarlo?, o por el contrario – ha estado tan inquieto(a) o agitado(a), que se ha estado moviendo mucho más de lo normal
