# Supplementary material for: Integrating psychosocial health into disaster risk management: Insights from COVID-19 in Durán, Ecuador
Source: PLoS One. 2026 Mar 27;21(3):e0343239. doi: 10.1371/journal.pone.0343239 (PMC13029789; doi:10.1371/journal.pone.0343239)
Supplement: S2 File — (PDF) [file pone.0343239.s002.pdf]

Oficio Nro. MSP-CGDES-2021-0209-O

Quito, D.M., 11 de agosto de 2021

Asunto: Aprobación del protocolo de investigación, codificado con el N° 107-201, titulado: "INSUMOS PARA UN SISTEMA DE ALERTA TEMPRANA PARA EL SARS-COV-2 (COVID-19): CASO PILOTO EN DURAN (INSAT COVID-19)".

Doctora
Mercy Julia Borbor Córdova
ESCUELA SUPERIOR POLITÉCNICA DEL LITORAL
En su Despacho

Con un atento saludo, hago alusión al documento MSP-DNSG-2021-17740-E, ingresado a la Coordinación General de Desarrollo Estratégico en Salud y enviado a los miembros del Comité Expedito el 13 de julio de 2021, con la finalidad de que el Comité creado mediante Acuerdo Ministerial 00003-2020, y que tuvo su reforma mediante acuerdo ministerial 00104-2020 del 8 de diciembre de 2020, realice la segunda evaluación del protocolo de investigación observacional en salud con uso de datos de historia clínica /uso de muestras biológicas humanas de pacientes infectados con SARS-CoV2 y/o con diagnóstico de COVID-19:

Titulado: “INSUMOS PARA UN SISTEMA DE ALERTA TEMPRANA PARA EL SARS-COV-2 (COVID-19): CASO PILOTO EN DURAN (INSAT COVID-19)”.

En este contexto anexo el informe realizado por los miembros del Comité Expedito, en el que indica que su protocolo de investigación codificado con el N° 107-2021, se encuentra aprobado, con recomendaciones verificables durante la ejecución del estudio, sin requerir verificación previa al inicio de su realización de acuerdo al cronograma presentado.

Cabe señalar que el Comité Expedito aprueba las investigaciones y enmienda en los aspectos éticos, metodológicos y jurídicos de los protocolos de investigación que soliciten una aprobación expedita; garantizando una evaluación ética, a fin de que las investigaciones se realicen bajo principios y valores éticos que garanticen la protección de la dignidad y los derechos de las personas y las comunidades.

Se recalca que los investigadores que hayan recibido la aprobación de ejecución del protocolo de investigación deberán remitir los reportes trimestrales de seguimiento de la investigación, así como un informe de finalización del estudio, a la Coordinación General de Desarrollo Estratégico en Salud, a la Dirección Nacional de Inteligencia de la Salud y al Viceministerio de Gobernanza y Vigilancia de la Salud, según lo estipula en el Acuerdo Ministerial 00104-2020 en su Art. 33.

Cabe indicar que el Art.34. del mismo Acuerdo Ministerial menciona: “Los investigadores que realicen los estudios que fueron aprobados bajo la revisión expedita de

acuerdo a las condiciones establecidas en el presente reglamento, deberán informar los productos intermedios del procesamiento de investigación y los resultados de sus investigaciones al Viceministerio de Gobernanza de la Salud y a la Coordinación General de Desarrollo Estratégico en Salud del Ministerio de Salud Pública".

Además, deberán difundir oportunamente los resultados obtenidos, a fin de que se constituyan como un aporte en la búsqueda de soluciones a este grave problema de salud pública.

Por otro lado, es necesario indicar que la Coordinación General de Desarrollo Estratégico en Salud, en base a las competencias y atribuciones otorgadas mediante "Estatuto Orgánico Sustitutivo de Gestión Organizacional por Procesos" del Ministerio de Salud Pública vigente no coordina o brindar apoyo logístico en temas de ejecución de proyectos de investigación, ni otorgar autorización sobre el uso de recursos humanos, materiales y/o financieros de los establecimientos del Ministerio de Salud Pública, para la ejecución de una investigación.

Además, se informa que las evaluaciones son procesadas secuencialmente, respetando la fecha de ingreso de los protocolos de investigación. Debido a la alta demanda de revisiones y evaluaciones de protocolos que ha tenido el Comité de Ética Ad Hoc creado por el Acuerdo Ministerial 00003-2020, para la Revisión Expedita de Investigaciones COVID-19, apelamos a su comprensión respecto al tiempo que se ha requerido para generar la respuesta correspondiente.

Aprovecho la ocasión para informar que: la máxima autoridad de esta cartera de estado, suscribió el Acuerdo Ministerial 00009-2021, del 28 de julio de 2021, en el cual se deroga el Acuerdo Ministerial No. 00104-2020, publicado en el Tercer Suplemento del Registro Oficial No.357 de 24 de diciembre de 2020, con el que se expidió el "Reglamento para la aprobación y desarrollo de investigaciones en salud relacionadas a COVID-19".

El nuevo Acuerdo Ministerial 00009-2021 en su Artículo 2.- indica: "Las investigaciones en salud que se planteen realizar en las personas con sospecha de COVlD19, en pacientes infectados por SARS-Co V-2, o en personas sanas, cuya participación se requiere para generar conocimiento relacionado con COVID-19, serán evaluadas y aprobadas por los Comités de Ética de Investigación en Seres Humanos
(CEISH) aprobados conforme al Acuerdo Ministerial No. 4889, publicado en el Suplemento del Registro Oficial No. 279 de julio de 2014; y, en caso de ensayos clínicos, según las disposiciones del "Reglamento para la aprobación, desarrollo, vigilancia y control de los ensayos clínicos" expedido con Acuerdo Ministerial No. 0075 de 30 de junio de 2017, publicado en la Edición Especial del Registro Oficial No. 23 de 30 de junio de 2017.

En este contexto se sugiere que, para su próximo estudio de investigación de COVID-19

lo pueda gestionar a través de un Comité de Ética en Investigación en Seres Humanos CEISH aprobado por el MSP, para lo cual podrá encontrar en el siguiente link la lista de los Comités de Ética de Investigación en Seres Humanos (CEISH) aprobado por esta cartera de estado:

https://www.salud.gob.ec/aprobacion-de-comites-de-etica/

En tal virtud y con la finalidad de dar cumplimiento a la normativa legal vigente y dar continuidad a los protocolos de investigación de COVID-19 la Dirección Nacional de Inteligencia de la Salud a través de la Coordinación General de Desarrollo Estratégico en Salud busca promover la investigación de calidad en el Ecuador.

Sin otro particular me despido, sin antes expresar mi sentimiento de consideración y alta estima.

Atentamente,

Documento firmado electrónicamente

Espc. Aquiles Rodrigo Henriquez Trujillo
COORDINADOR GENERAL DE DESARROLLO ESTRATÉGICO EN SALUD

Referencias:
- MSP-DNSG-2021-17740-E

Anexos:
- informe_de_aprobacion_107-2021.pdf

Copia:

Señor Doctor
Víctor Manuel Pacheco Bastidas
PRESIDENTE DE LA COMISIÓN NACIONAL DE BIOÉTICA EN SALUD

Señorita Doctora
Katherine Lizeth Simbaña Rivera
Directora Nacional de Inteligencia de la Salud

Señora Magíster
Maribel Del Rocío Rhon Bunshi
Especialista de Evaluación de Tecnología Sanitaria 1

Señorita Técnica
Laura Mercedes Torres Heredia
Asistente Administrativa 3

Oficio Nro. MSP-CGDES-2021-0209-O

Quito, D.M., 11 de agosto de 2021

lt

Firmado electrónicamente por:
AQUILES RODRIGO HENRIQUEZ TRUJILLO

Official Document No. MSP-CGDES-2021-0209-O

Quito, D.M., August 11, 2021

Subject: Approval of the research protocol, coded with No. 107-201, titled: "SUPPLIES FOR AN EARLY WARNING SYSTEM FOR SARS-COV-2 (COVID-19): PILOT CASE IN DURAN (INSAT COVID-19)".

To:

Dr. Mercy Julia Borbor Córdova

ESCUELA SUPERIOR POLITÉCNICA DEL LITORAL

At your office

Dear Dr. Borbor,

With respectful regards, I refer to the document MSP-DNSG-2021-17740-E, received by the General Coordination of Strategic Health Development and sent to the members of the Expedite Committee on July 13, 2021. The purpose of the document was for the Committee, established by Ministerial Agreement 00003-2020, and reformed by Ministerial Agreement 00104-2020 on December 8, 2020, to conduct the second evaluation of the observational health research protocol using clinical history data and/or biological samples from patients infected with SARS-CoV-2 and/or diagnosed with COVID-19:

Titled: "SUPPLIES FOR AN EARLY WARNING SYSTEM FOR SARS-COV-2 (COVID-19): PILOT CASE IN DURAN (INSAT COVID-19)".

In this context, I attach the report prepared by the members of the Expedite Committee, indicating that your research protocol, coded with No. 107-2021, is approved with recommendations to be verified during the execution of the study, without requiring prior verification before the start of its execution according to the schedule presented.

It should be noted that the Expedite Committee approves research and amendments in the ethical, methodological, and legal aspects of research protocols that request expedited approval, ensuring an ethical evaluation to guarantee that research is conducted under

principles and ethical values that protect the dignity and rights of individuals and communities.

It is emphasized that researchers who have received approval for the execution of the research protocol must submit quarterly monitoring reports of the research, as well as a final study report, to the General Coordination of Strategic Health Development, the National Health Intelligence Directorate, and the Vice Ministry of Health Governance and Surveillance, as stipulated in Article 33 of the Ministerial Agreement 00104-2020.

It should be noted that Article 34 of the same Ministerial Agreement states: "Researchers conducting studies approved under the expedited review according to the conditions established in this regulation, must report the intermediate products of the research process and the results of their research to the Vice Ministry of Health Governance and the General Coordination of Strategic Health Development of the Ministry of Public Health."

Furthermore, they must promptly disseminate the results obtained so that they can contribute to finding solutions to this serious public health problem.

On the other hand, it is necessary to indicate that the General Coordination of Strategic Health Development, based on the competencies and attributions granted by the "Substitute Organic Statute of Organizational Management by Processes" of the Ministry of Public Health in force, does not coordinate or provide logistical support on issues of project execution, nor grant authorization for the use of human, material, and/or financial resources from the Ministry of Public Health establishments for the execution of research.

Additionally, it is reported that evaluations are processed sequentially, respecting the date of entry of research protocols. Due to the high demand for reviews and evaluations of protocols that the Ad Hoc Ethics Committee (created by Ministerial Agreement 00003-2020 for Expedited Review of COVID-19 Research) has received, we appeal to your understanding regarding the time required to generate the corresponding response.

I take this opportunity to inform you that the highest authority of this state portfolio signed Ministerial Agreement 00009-2021, dated July 28, 2021, which revokes Ministerial Agreement No. 00104-2020, published in the Third Supplement of Official Register No. 357 of December 24, 2020, which issued the "Regulation for the approval and development of health research related to COVID-19."

The new Ministerial Agreement 00009-2021 states in Article 2: "Health research proposed to be conducted on individuals suspected of having COVID-19, in patients infected by SARS-CoV-2, or in healthy individuals whose participation is required to generate knowledge related to COVID-19, will be evaluated and approved by the Ethics Committees for Research on Human Beings (CEISH) approved under Ministerial Agreement No. 4889, published in the Supplement of Official Register No. 279 of July 2014; and, in the case of clinical trials, according to the provisions of the 'Regulation for the approval, development, surveillance, and control of clinical trials' issued by Ministerial Agreement No. 0075 of June 30, 2017, published in the Special Edition of Official Register No. 23 of June 30, 2017."

In this context, it is suggested that for your next COVID-19 research study, you manage it through an Ethics Committee for Research on Human Beings (CEISH) approved by the MSP, for which you can find the list of the Ethics Committees for Research on Human Beings (CEISH) approved by this state portfolio at the following link:

https://www.salud.gob.ec/aprobacion-de-comites-de-etica/

Therefore, and to comply with the current legal regulations and ensure the continuity of COVID-19 research protocols, the National Health Intelligence Directorate, through the General Coordination of Strategic Health Development, seeks to promote quality research in Ecuador.

With no further comments, I bid you farewell, expressing my consideration and high esteem.

Sincerely,

Espc. Aquiles Rodrigo Henriquez Trujillo

GENERAL COORDINATOR OF STRATEGIC HEALTH DEVELOPMENT

References:

- MSP-DNSG-2021-17740-E

Attachments:

- approval_report_107-2021.pdf

Cc:

Dr. Víctor Manuel Pacheco Bastidas

PRESIDENT OF THE NATIONAL BIOETHICS COMMISSION IN HEALTH

Dr. Katherine Lizeth Simbaña Rivera

National Health Intelligence Director

Ms. Maribel Del Rocío Rhon Bunshi

Health Technology Assessment Specialist 1

Ms. Laura Mercedes Torres Heredia

Administrative Assistant 3
