# Supplementary material for: Integrating psychosocial health into disaster risk management: Insights from COVID-19 in Durán, Ecuador
Source: PLoS One. 2026 Mar 27;21(3):e0343239. doi: 10.1371/journal.pone.0343239 (PMC13029789; doi:10.1371/journal.pone.0343239)
Supplement: Attachment — Submitted filename: Reply_to_Reviewers_PONE-D-24-34167_rev (1).docx [file pone.0343239.s004.docx]

Reviewer # 1

Comment / Answer to comment / Lines/Pages in manuscript

1. Subheading 3.1 - The statistical methods should be explained with procedures, interpretations, and equations/formulas.

This has been added.
In the statistical analysis section, we added a complete explanation of the correlation and regression analysis with the formula required. We eliminated numbering headings.

Lines 256-294.

2. Discuss these articles which focus on disaster vulnerability.

https://link.springer.com/article/10.1007/s10668-023-04149-1

https://link.springer.com/article/10.1007/s00477-022-02267-2

Thank you for the references on disaster vulnerability factors, we have included them in the Introduction section

Reference 37 and 38

Lines 119 -121 - 515

3. The subheading numbering should be properly arranged.

We have rearranged the headings, subheadings that were edited to adhere to the journal guidelines. We eliminated the numbering.

In the whole document.

4. The results should be justified with the support of relevant published articles.

Thank you for your comment. The interpretation of the results with relevant published articles were included in the discussion.

Lines 432 -517

5. The influence of all variables (mentioned in the Tables) on disaster vulnerability should be explained in the Methods section.

The Methods/Survey is under the subsection "Vulnerability Framework Variables", it explains the vulnerability framework, and the variables that are related to the impacts of mental health.

Lines 203 - 216

6. Improve the quality of English to rectify the typo and grammatical errors.

The manuscript has been revised and corrected for typos and grammar.

In the whole document.

7. Keywords - Include only 5 keywords which are relevant.

The manuscript now has just 5 keywords

Lines 65 - 66

**Reviewer # 2**

**Comment / Answer to comment /Lines/Pages in manuscript**

1. Photos are not clear enough

Diagrams were converted to TIIF format to improve the resolution and integration in the word document.

Attach as tiff files

2. Please give a brief description of the study area Duran such as population, economy, orientation, etc. to help the reader understand it

We have added a concise description of Duran's main characteristics in the Study Area subsection.

Lines 145-153

3. The introduction is too long and not very relevant.

Thank you for your comment to improve our Introduction: We have restructured the Introduction to incorporate the most relevant concepts related to psychosocial health and Disaster Risk Management (DRM), highlight the research problem concerning the lack of integration between these two fields, and explain why this integration is particularly important in our region. We also emphasize the usefulness of this type of research for decision-makers and strategy implementation. We hope that the revised Introduction will better guide the reader through the rest of the paper.

Lines 67 -142

4. The methodological content is too simple and some necessary methodologies are not considered.

The methodological content has been expanded in the Methods section, including statistical analysis to clarify the methodological process.

Lines 144 - 244

5. 221-223 grading criteria please add references.

The scoring has been described in each Measure (PSS-10 and PHQ-9) subheading. We have added the References: Ruisoto P, López-Guerra VM, Paladines MB, Vaca SL, Cacho R. Psychometric properties of the three versions of the Perceived Stress Scale in Ecuador. 2020; Available from: <https://doi.org/10.1016/j.physbeh.2020.113045>

Carroll HA, Hook K, Perez OFR, Denckla C, Vince CC, Ghebrehiwet S, et al. Establishing Reliability and Validity for Mental Health Screening Instruments in Resource-Constrained Settings: Systematic Review of the PHQ-9 and Key Recommendations. Psychiatry Res. 2020;291:113236. doi: 10.1016/j.psychres.2020.113236

Ref 54, 57

Lines 228-254

6. P-value should be italicized.

Thank you. This has been updated in the tables

Tables 2,3,4

7. How was the independent variable selected?

The independent variables were selected based on the literature on psychosocial vulnerability and adaptive capacity in the context of disasters and health crises. Specifically, individual and urban sensitivity variables identified in our previous studies (references 2, 3, 26, 29, 31, 43 of the manuscript). See Vulnerability Framework subheading

Lines 202 -225

8. Is OR corrected and how are confounders considered?

We thank you for your thoughtful contribution. To control for confounding, we employed a two-stage approach. First, as a precursor to model selection, we tested how strong the association between stress and depression level and a particular predictor, employing appropriate measures of association between categorical data —Kendall’s Tau-b, Tau-c, and Cramér’s V (Table 2). With these coefficients, we selected which predictors to include in the model. Second, because stress and depression levels had many categories, we employed a multinomial logistic regression model. Employing this methodology, we estimated adjusted odds ratios for various covariates. Comparing crude and adjusted ORs, we identified which had confounding effects—those that altered association magnitude or direction. This modeling technique confirmed that observed associations reflect independent effects and controlled for confounding impact of other applicable covariates. Multinomial logistic regression provided a statistically rigorous basis for the investigation of the contribution of a particular predictor to stress and depression levels. See subheading Regression analysis

Lines 273 - 274

9. How was sample size calculation considered?

In non-probabilistic sampling, it is not appropriate to apply measures designed for probability-based sampling to assess representativeness (Baker et al. 2013). The non-probabilistic method used in Duran’s study was based in the representativeness of system (Duran city), by selecting sites based on in-depth knowledge of Duran’s urban structure and social vulnerability (Borbor-Cordova et al., 2020). Additionally, the use of the multinomial logistic regression model enhances the robustness of our findings, considering that it is suitable for analyzing complex relationships among multiple variables (vulnerability variables), even with non-probabilistic sampling. We argue that the deliberate selection of sites—based on contextual knowledge of urban structure, social vulnerability, and —was essential in mitigating bias during survey administration. See subheading Study design and settings.

Baker R, Brick JM, Bates NA, Battaglia M, Couper MP, Dever JA, et al. Summary report of the AAPOR task force on non-probability sampling. J Surv Stat Methodol. 2013 Nov 1;1(2):90–105

Lines 155 -175

**Reviewer #3**

**Comment / Answer to comment / Lines/Pages in manuscript**

Introduction

Please bring the following items:

- Definition of the research problem

- The magnitude and importance of the study variable

- Expressing the necessity of conducting the study

- Finally, the practical purpose of the study should be stated.

We have restructured the Introduction to include the most relevant concepts related to Psychosocial health and Disaster Risk Management, the research problem related to the lack of integration of these approaches, why is relevant in the context of cities and the usability this kind of research for decision makers and implementing strategies at city level. We hope this Introduction will guide the reader into the paper.

Lines 67 - 78

Lines 91 -100

Lines 115-121

Method

1. The method section should include the sample size formula.

In non-probabilistic sampling, it is not appropriate to apply measures designed for probability-based sampling to assess representativeness (Baker et al. 2013). The non-probabilistic method used in Duran’s study was based in the representativeness of system (Duran city), by selecting sites based on in-depth knowledge of Duran’s urban structure and social vulnerability (Borbor-Cordova et al., 2020). Additionally, the use of the multinomial logistic regression model enhances the robustness of our findings, considering that it is suitable for analyzing complex relationships among multiple variables (vulnerability variables), even with non-probabilistic sampling. We argue that the deliberate selection of sites—based on contextual knowledge of urban structure, social vulnerability, and —was essential in mitigating bias during survey administration. See Study design and settings.

Baker R, Brick JM, Bates NA, Battaglia M, Couper MP, Dever JA, et al. Summary report of the AAPOR task force on non-probability sampling. J Surv Stat Methodol. 2013 Nov 1;1(2):90–105

Lines 155 -175

2. Exclusion and inclusion criteria are fully stated.

The inclusion and exclusion criteria are stated in the subsection "Study design and settings."

Lines 159-161.

3. The author did not mention any incomplete, incorrect or corrupt questionnaire

Out of the 446 questionnaires, we excluded those that did not have completed the questionnaires on stress and depression, ending with 340. See subsection "Study design and settings" for description

Lines 171-175

4. The first paragraph of the discussion should be the overall result of the study.

Thank you for your comment, the first paragraph states the overall results of the study.

Lines 432 - 441

5. Did the questionnaire undergo any changes in its questions post the validation and reliability test? Were any questions removed or added? Please provide clarification.

The questionnaire remained unchanged after the validation and reliability tests, with no items added or removed. The final version used in the study was fully consistent with the validated instrument. Validation details are included in the supplementary material (PSS-6 and PHQ-9) for transparency. See section Measures.

Line 227

Conclusion

1. What are the strengths and limitations of the study?

Thank you for your comments, we have added the strengths and explain the limitations of the study. See the Strengths and Limitations section

Lines 576 - 588

2. What are your suggestion for future studies?

We have included them in section 'Conclusions and future work'.

Lines 590 – 621

**Reviewer #4**

**Comment / Answer to comment /Lines/Pages in manuscript**

1. Any restrictions on data sharing should be specified.

We appreciate the reviewer’s attention to data sharing compliance. In alignment with Ecuador’s Organic Law on Data Protection (Ley Orgánica de Protección de Datos Personales, 2021), we have ensured that our data handling practices adhere to national regulations and the approval of the ethics committee. Thus, we have prepared an anonymized dataset that complies with national data protection standards and is now available for public sharing.

DOI: 10.6084/m9.figshare.28784126

2. It is also suggested that the article elaborate more on the social and economic impacts of COVID-19 on mental health.

In the subsections of the Discussion, we have elaborated more on the social and economic impacts of COVID-19 on mental health, supported by references from global and local examples.

Lines 443 - 517

3. More detail could be provided on sample selection to address potential biases due to non-probabilistic sampling.

This has been added in subheading Study design and settings.
We would like to provide clarification regarding our sampling methodology: due to logistical constraints imposed by the COVID-19 pandemic, we employed a non-probabilistic convenience sampling approach for this study. To mitigate potential biases associated with this method, we conducted outreach campaigns across various urban sectors, aiming to maximize the diversity of our sample. Furthermore, we compared the sociodemographic characteristics of our participants with official population data from Durán to assess partial representativeness.

While we acknowledge that non-probabilistic sampling limits the generalizability of our findings, such approaches are often necessary in exploratory research, especially under challenging circumstances like a pandemic. This methodology allowed us to gain a better understanding of the Duran vulnerability to disaster, which can inform future, more comprehensive studies.

Lines 155 - 175

4. Please provide more information about handling missing or outlier data to increase accuracy.

This has been included.
Missing values in our dataset were minimal. The variable with the most missing values had only 12 out of 340 cases (<3.5%). Due to the low proportion of missing data and following standard practice, imputation was not performed as it would have had minimal impact on the estimates.

We performed the multinomial logistic regression using listwise deletion. This did not affect the significance or interpretation of the results, and parameter estimates remained stable. We, therefore, concluded that missing data did not introduce bias or compromise the robustness of the findings.

No extreme outliers were identified in key variables. Visual inspection and statistical checks confirmed the appropriateness of data distributions before model fitting.

5. The manuscript is generally clear and written in standard English. Minor typographical and grammatical errors exist (e.g., “stress and depression of volunteers aiming” could be clearer), and some sections (e.g., the Methods) could be streamlined for conciseness. A proofreading step before submission is recommended.

This has been resolved. We have revised the whole manuscript for typos and grammar, and we have focused on clarity and conciseness.

All the manuscript

6. Reorganize the Results section to highlight key findings concisely, avoiding redundancy with tables.

Results have been reorganized to highlight the key findings, in subsections of stress and depression.

Lines 349 - 421

7. Expand the discussion on how the findings inform future disaster recovery policies, specifically within urban contexts similar to Duran

In the Discussion subsections we have addressed how the findings inform future recovery by building resilient communities and fostering integrated policy within the urban context using the case of Duran.

Lines 519 - 553

8. Provide clearer links between psychosocial findings and broader systemic risks like climate change.

The section titled "Psychosocial factors and systemic vulnerability" was restructured in the “Urban vulnerability” in which the systemic risk has been addressed.

Lines 502 - 517

9. A broader description of forward-looking policies: The discussion could elaborate more on how the findings could be implemented in future disaster management and reconstruction plans, especially in cities with similar vulnerability structures.

Thank you for your recommendations that has been included in some subsections of the Discussion, Conclusions and Future work.

Lines 590 - 621

10. Emphasis on practical solutions: The discussion could focus more on practical solutions and policy suggestions for reducing stress and depression in vulnerable populations.

This has been included in Discussion subsections of A Pathway to Building Resilient Communities and Integrating Health Care, Mental Health Systems, and Disaster Risk Management

Lines 519 – 574

**Reviewer #5:**
 Thank you for your well performed study. The research is methodologically sound, and the integration of psychosocial health into disaster risk management is a commendable approach that can fill a significant gap in disaster preparedness literature. The findings related to gender, housing conditions, and chronic illnesses offer a critical perspective on psychosocial vulnerability in disaster contexts.

Thank you for your positive feedback and for highlighting the importance of integrating psychosocial health and factors such as gender, housing, and chronic illness into disaster risk management.

**Reviewer # 6
Comment / Answer to comment Lines/Pages in manuscript**

1. Introduction

- Line 68-181: While the introduction contains important evidence about COVID-19, psycho-social health, it also includes excessive descriptions that make it less focused.

We have rewritten to streamline the Introduction, accordingly, following your suggestions

Line 67 - 142

2. Some parts of the introduction appear unrelated to the research topic. For example, the integration of psychosocial health into disaster risk management is inadequately described and lacks sufficient context about Ecuador’s specific situation. I would suggest streamlining this section and ensure that all the introduction content directly supports the study’s objectives.

Thank you for your insights that have helped us to improve our manuscript. We have integrated psychosocial health into disaster risk management in the context of Ecuador and valid for similar contexts in the Latin American region.

Lines 87 - 100

3. Line 183: The calculation of the sample size, the sampling distribution, and sampling representativeness of the study population are not clearly outlined in the methodology.

See section Study design and settings:

This has been addressed, we would like to provide clarification regarding our sampling methodology: due to logistical constraints imposed by the COVID-19 pandemic, we employed a non-probabilistic convenience sampling approach for this exploratory study. To mitigate potential biases associated with this method, we conducted outreach campaigns across various urban sectors, aiming to maximize the diversity of our sample. Furthermore, we compared the sociodemographic characteristics of our participants with official population data from Durán to assess partial representativeness.

While we acknowledge that non-probabilistic sampling limits the generalizability of our findings, such approaches are often necessary in exploratory research, especially under challenging circumstances like a pandemic. This methodology allowed us to gain a better understanding of the Duran vulnerability to disasters which can inform future, more comprehensive studies.

Lines 155 - 163

4. Line 188-194: The study population under the methodology part describes about collection of blood samples and the use of non-probability sampling to select participants, however it does not adequately introduce the study area and population. I would suggest a clearer explanation of where the study was conducted and the characteristics of the study population.

Thank you for your comment. We have addressed your comment in the subsections Study Area and Study design, and settings.

Lines 145 - 153

Lines 155 – 179

5. Line 196 – 213: The term “survey” is not clearly defined in the methodology. The description appears to mix different components of the methods part, including data collection, data collection tools, and other aspects. This makes it difficult for readers to determine which aspect of the methodology is being discussed.

Under Methods section we have included now a subsection titled Survey to address your concern.

Lines 181 - 200

6. Line 234: The statistical categorization and analysis used for the modified Spanish version of the Perceived Stress Scale (PSS) and the Patient Health Questionnaire-9 (PHQ-9) need to be explicitly detailed and a clearer explanation of how these tools were analyzed would strengthen the methodological rigor of the study.

Your critical insight has been addressed in the subsections Statistical Analysis, Correlation Analysis, and Regression Analysis.

Lines 257 - 259

Lines 267 - 271

Lines 274 – 294

7. Line 257: The process by which the 340 participants were selected to complete the survey is not clearly described.

We have addressed your concern, and we now have a complete explanation about the 340 participants in the 'Study design and settings' subsection

Lines 171 -175

8. Line 265: The terminology used to describe educational levels, such as “some level of high school education,” “some level of elementary school,” and “some level of undergraduate studies,” appears subjective and lacks precision.

We have addressed your concerns, and we have changed them into adequate and proper wording in the four tables and results.

Lines 310 -312
Lines 345 - 347
Lines 377 - 381
Lines 401 - 403
Lines 423 - 424

9. Line 269: Some variables presented in the table, such as education and marital status, sum precisely to 100%. The table should be checked for consistency to ensure accuracy.

Thank you for your comments. We have revised and added to the table options, the " No answer" response to ensure accuracy.

Lines 345 – 347

10. Line 342: The results section includes a discussion of self-reported stress, which is valuable. However, the methods section should explicitly describe under the methodology part how self-reported data were analyzed.

Thank you for your relevant commentary, we have expanded the Methods section to include the Statistical analysis subsections of Correlation Analysis, and Regression Analysis.

Lines 257 - 259

Lines 267 - 271

Lines 274 – 294

11. Line 376: To improve the identification of risk factors and the prediction of stress levels, I would recommend to include adjusted odds ratio (AOR) results in addition to crude odds ratio (COR) results. This would provide a more comprehensive understanding of the associations between variables after adjusting for potential confounding variables.

We thank you for your thoughtful contribution. To control for confounding, we employed a two-stage approach. First, as a precursor to model selection, we tested how strong the association between stress and depression level and a particular predictor, employing appropriate measures of association between categorical data —Kendall’s Tau-b, Tau-c, and Cramér’s V (Table 2). With these coefficients, we selected which predictors to include in the model. Second, because stress and depression levels had many categories, we employed a multinomial logistic regression model. Employing this methodology, we estimated adjusted odds ratios for various covariates. Comparing crude and adjusted ORs, we identified which had confounding effects—those that altered association magnitude or direction. This modeling technique confirmed that observed associations reflect independent effects and controlled for the confounding impact of other applicable covariates. Multinomial logistic regression provided a statistically rigorous basis for the investigation of the contribution of a particular predictor to stress and depression levels.

Lines 401 - 404

**Reviewer # 7**

**Comment / Answer to comment / Lines/Pages in manuscript**

1. The manuscript states that a non-probabilistic sampling strategy was used. However, it does not explain how this might have affected the generalizability of the results. The implications of this method for bias and generalizability were not found. The authors should justify why this method was chosen and discuss its limitations.

See section Study design and settings:

This has been addressed, We would like to provide clarification regarding our sampling methodology: due to logistical constraints imposed by the COVID-19 pandemic, we employed a non-probabilistic convenience sampling approach for this exploratory study. To mitigate potential biases associated with this method, we conducted outreach campaigns across various urban sectors, aiming to maximize the diversity of our sample. Furthermore, we compared the sociodemographic characteristics of our participants with official population data from Durán to assess partial representativeness.

While we acknowledge that non-probabilistic sampling limits the generalizability of our findings, such approaches are often necessary in exploratory research, especially under challenging circumstances like a pandemic. This methodology allowed us to gain a better understanding of the Duran vulnerability to disasters which can inform future, more comprehensive studies.

Lines 155 - 163

2. The recruitment process lacks details about how participants were approached and selected, which is critical for assessing potential biases in the sample. Explain how the sample represents the broader population of Duran.

Thank you for your comment. We have explained this in subsection 'Study design and settings'

Lines 155 - 179

3. Also, provide evidence/references that the Perceived Stress Scale (PSS) are validated/previously used for the Ecuadorian population?

This reference and the following sentence were added to the PSS section of the paper: https://www.sciencedirect.com/science/article/abs/pii/S0031938420303590 "The PSS-10 and PSS-4 have been recommended over the PSS-14 for use in Ecuador." Ref 55, Ruisoto P, López-Guerra VM, Paladines MB, Vaca SL, Cacho R. Psychometric properties of the three versions of the Perceived Stress Scale in Ecuador. 2020 [cited 2025 Mar 2]; Available from: <https://doi.org/10.1016/j.physbeh.2020.113045>

Lines 829 -830

4. The manuscript does not state whether potential confounders were adjusted for in the logistic regression models. Given that multiple factors influence mental health (e.g., socioeconomic status, education, pre-existing conditions, vaccination status), were adjustments made?

We thank you for your thoughtful contribution. To control for confounding, we employed a two-stage approach. First, as a precursor to model selection, we tested how strong the association between stress and depression level and a particular predictor, employing appropriate measures of association between categorical data —Kendall’s Tau-b, Tau-c, and Cramér’s V (Table 2). With these coefficients, we selected which predictors to include in the model. Second, because stress and depression levels had many categories, we employed a multinomial logistic regression model. Employing this methodology, we estimated adjusted odds ratios for various covariates. Comparing crude and adjusted ORs, we identified which had confounding effects—those that altered association magnitude or direction. This modeling technique confirmed that observed associations reflect independent effects and controlled for confounding impact of other applicable covariates. Multinomial logistic regression provided a statistically rigorous basis for the investigation of the contribution of a particular predictor to stress and depression levels.

Lines 401 - 404

5. Provide goodness-of-fit tests and discuss whether multicollinearity was assessed. The study finds that women are more likely to experience depression. However, other factors (such as housing conditions, chronic illnesses, or socioeconomic status) could also contribute to depression.

Thank you for your insightful comment on the model’s goodness-of-fit. To evaluate fit in our multinomial logistic regression model, we assessed model deviance and conducted a likelihood ratio test comparing the full model to a nested baseline. We have not reported the deviance. However, the modest reduction in deviance indicated a limited but expected improvement in fit, given the complexity of the psychological outcome and the number of predictors included.

Regarding factors associated to depression, we have added in the discussion other factors that contribute to depression supported by references.

Lines 290 - 294

6. Causal Language Should Be Avoided. Phrases such as “vaccination acted as a protective factor against depression” should be reworded to reflect association rather than causation.

Thank you for your suggestion, we have reworded the text in the results section to reflect association rather than to causation.

**Lines 410 - 413**

7. Provide a deeper comparison with global and regional studies, particularly in Latin America.

Thank for your suggestion, in the Discussion section we have added papers that considered the global and Latin American context pertinent to our study. Will also add: https://pubmed.ncbi.nlm.nih.gov/35438066/ https://pmc.ncbi.nlm.nih.gov/articles/PMC8785692/ (Lines 415-600 whole discussion).

Lines 432 - 517

**Reviewer # 8**

**Comment / Answer to comment / Lines/Pages in manuscript**

1. The UNDRR has adopted the concept of "Disaster" to include pandemics and infectious diseases rather than focusing solely on natural disasters. The Sendai Framework for Disaster Risk Reduction, established in 2015, reflects discussions from the Convention on the Rights of Persons with Disabilities and emphasizes the growing collaboration between the health and welfare sectors and disaster sectors. The boundary between disasters and pandemics has virtually disappeared. However, why should we revisit the discussion in this manuscript about integrating psychosocial health with disaster risk management strategies?

Thank you very much for your critical insights We revisit the discussion in this manuscript about integrating psychosocial health with disaster risk management strategies for several reasons: In spite of the existence of the Sendai Framework, in the Global South these processes are not fully integrated yet, due to lack of knowledge, institutional fragmentation, limited capacities and funding. Therefore, to analyze the specific context is relevant to establish the measures for breaking these barriers and fill the gaps for the integration of DRM, Health and Disabilities within Latin American cities

Lines 87 - 100

Lines 538 – 553

2. Although this study is titled “Integrating Psychosocial Health into Disaster Risk Management,” it primarily focuses on examining the mental health effects of the COVID-19 pandemic. There is minimal mention of the connection between theories and practices of disaster risk reduction. Therefore, the title should be revised or the argument adjusted to better align with the title.

Thank you for your critical insight. The argument has been aligned with the title, with the DRM processes focusing on the preparation and recovery in the specific context of Latin American cities.

3. It does not consistently adhere to the specified styles and formatting. This includes the order of manuscript organization, abstract, numbering, and font size of sections and sub-sections, as well as citing references.

- Abstracts should be limited to 300 words and are usually unstructured.

- Cite references using square brackets instead of parentheses (for example, “[1]”).

- Include page numbers.

- These are just a few examples; the document needs significant revision.

Abstract was reformatted to be unstructured, page numbers were added (whole manuscript), formatting was edited to align with journal guidelines (whole manuscript).

We have added different examples global, regional, and local in the introduction and discussion.

References were updated, and the text citations use brackets now instead of parentheses, following the journal. guidelines

Lines 39 - 63

4. Throughout history, humans have faced numerous pandemics, including HIV, the Hong Kong flu (H3N2 influenza A virus), SARS, and MERS since the late 20th century. Shouldn't we consider the effects of these past pandemics on psychosocial health in different regions during the discussion of this study?

In this study we have focus on the integration of DRM with mental health, however, we have mentioned the other pandemics and its effects in the Introduction session.

Lines 97 - 100

5. It is crucial to emphasize the importance of the implementation in Ecuador, the universal nature of the results, their general and specific aspects, and the regional context surrounding these outcomes. As you know, people's health and behavior are closely connected to the target region's culture, customs, history, politics, socioeconomic background, and social institutions. Therefore, comparing one country to another or applying findings from one context to a different one may not be suitable. The discussion should differentiate between the regional and universal aspects of the results and then clarify the significance of what this study has uncovered. This also pertains to the novelty and originality of the study.

We agree with your comments, and we have incorporated your ideas into the Discussion section with several subsections: Socio-cultural Vulnerability, Health related vulnerability, and Urban Vulnerability. We have reviewed the universal and local aspects using scientific references and also taking into consideration our own knowledge from the long-term partnership with the Duran city and its communities.

Lines 443 - 481

Lines 483 - 500

Lines 502 – 517

6. Figure 1 must be adequately explained in the study area sub-section. This explanation should cover the location, population, urban environment, and social and family systems, among other things. Additionally, it should convey to the reader why this area is most suitable for this study. Regardless, the absence of text in the provided subsection is a significant issue. (Line 183-187)

Thank you for your critical and valid observation, We have added in the Methods section the following subsections: study area, Study design and settings and the Vulnerability framework variables to address this issue.

Lines145 - 153

Lines 156- 170

Lines 203 – 216

7. Figures should be appropriately cited and described in the text. (Figure 1 and 3)

Figures 1 and 3 have been described in the text and appropriated cited.

Lines 170 - 177

Lines 216 – 225

8. Multiple surveys are presented, creating the impression of a composite of several studies. Why are different surveys necessary, and what is their connection? For instance, a sub-section on blood sample collection before the "2.1 Survey" sub-section, without any reference to the relationship or necessity between the two, contributes to confusion. Sometimes, it may be necessary to divide the paper into several parts.

Thank you to the reviewer for your observation, we have clarified in the section Survey the process and the variables in the vulnerability framework

Lines 181 - 195
Lines 202 - 216
Lines 227 - 254

9. The section titles and setting sections require revisions to improve their suitability. For example, the sub-sections "2.1 Survey,” "2.2 Perceived Stress Scale," and “2.3 Patient Health Questionnaire-9 for Depression” should not maintain a parallel structure.

Thank you for your observation. We have reviewed the section and subsection titles

Lines 181 - 195
Lines 202 - 216
Lines 227 - 254

10. It is unclear which surveys and analysis methods are associated with which results.

Thank you for your observation. We have revised the Methods section to clearly indicate which survey modules and analysis techniques correspond to each set of results.

Line 303
Line 349
Line 405

11. What does this research reveal as the most significant new finding? Aren't all the at-risk individuals mentioned in the results those who have long been considered vulnerable in many studies? The abstract highlights that women experienced more significant psychosocial impacts; however, are these results different from those of the other countries mentioned in the introduction?

Thank you for your thoughtful question. While our findings align with existing literature identifying women and individuals with chronic illnesses as vulnerable groups, our study offers a novel contribution by demonstrating that the same vulnerability factors relevant to other hazards, such as inadequate housing and limited access to essential services, also significantly influence psychosocial outcomes during pandemics. This confirms the need for integrated disaster risk management and public health policies that prioritize reducing vulnerabilities.

Lines 502 -517

Lines 538 -553

12. In the Discussion, it is essential to provide a scientifically valid interpretation based on the statistical trends and correlations found in the Results. This should include insights from previous studies in epidemiology, social sciences, and the humanities (these studies should be referenced in the introduction) and information about the study area.

Thank you for your critical insight. We have addressed your concerns in the Discussion, with the interpretation of the results and supported by references of previous epidemiological, and interdisciplinaries studies. We also have added the information of the study area.

Lines 145-153

Lines 443 - 481

Lines 483 – 500

13. It is essential to emphasize pre-disaster preparedness, as indicated by the term DRR (Disaster Risk Reduction) in the SFDRR. A categorized list of the study's findings and identified countermeasures, organized by disaster phase and target attribute, would enhance understanding.

This is the reason why we choose the vulnerability framework presented in the paper. Within the Discussion we have identified the study findings and in the subsections 'A Pathway to Building Resilient Communities' and 'Integrating Health Care, Mental Health Systems, and Disaster Risk Management' we have identified several countermeasures. We have focused on two phases: preparedness and recovery ones.

Lines 519 - 553

Lines 555 – 574

14. How can perceptions of stress be lower among those experiencing high stress levels? At a minimum, what do the results of this study reveal about the challenges, blind spots, and bottlenecks in policies and efforts during the COVID-19 pandemic?

We have not explored the psychological variables in depth; we have focused on the relationships between mental health and disaster risk management in urban settings, market by vulnerability and systemic inequalities. This is why we have recommended future research (within the section Conclusions and future work) to focus on key areas to strengthen the integration of psychosocial health within disaster risk management, such as:

• Multi-hazard Early Warning Systems (EWS) and community engagement would be key in this goal.

• Building up a database of psychological impacts along with the assessment of local mental health interventions,

Lines 605 -619

15. If the results indicate that married women are at a significantly higher risk than others, it is crucial to explore which policies and support measures could effectively address their needs based on the findings. What should the family, community, religion, and government have done?

We have talked about women at higher risk, considering our local cultural context on how the city works. This allows us to recommend the work on increasing women's adaptive capacity. Actions such as Literacy program for women, empowering programs for women and girls, trust development in the available services.

Lines 547 - 553

16. Why are participants living in areas without paved roads experiencing higher stress levels (due to social and environmental factors)? Is it reasonable to classify residents of areas without paved roads as informal settlers? What is the reasoning behind this?

We have explained this concept under sensitivity variables. Yes, informal settlers are an important factor that is mentioned in the introduction and discussion. Reasons are related to the lack of services, infrastructure, and housing conditions. This is explained in the Urban vulnerability subsection.

Lines 502 - 517

17. The reasons for elevated stress levels among those living in rental apartments require a more thorough explanation. What types of living environments, price ranges, and occupations do rental apartments in the target area typically accommodate, and how do these compare to other housing options?

Thank you for your observation. We added in the discussion a subsection of Urban Vulnerability where we address some if the issues of housing and informality supported by global and regional references.

Lines 502 - 517

18. A sub-section titled "Moving forward" should not be included as a subsection at the beginning of the Discussion. Instead, it should be placed at the end of the conclusion, depending on the content of the Discussion, if you wish to include it.

There is a new subsection in the Discussion section, under the title "A Pathway to Building Resilient Communities' and also in the conclusions.

Lines 519 - 553

Lines 601 -620

**Reviewer # 9**

**Comment / Answer to comment / Lines/Pages in manuscript**

Certain areas require further clarification and enhancement. To enhance the manuscript, read and cite the following related articles in the discussion:

- https://doi.org/10.3390/ijerph18115719
- https://doi.org/10.3389/fpsyt.2021.656664
- https://doi.org/10.1017/dmp.2021.140
- https://doi.org/10.1371/journal.pone.0280144
- https://doi.org/10.1080/13623699.2021.1950519 https://www.thelancet.com/journals/lanpsy/article/PIIS2215-0366(24)00354-7/abstract

These have been included.
https://doi.org/10.3390/ijerph18115719

https://doi.org/10.3389/fpsyt.2021.656664

https://doi.org/10.1017/dmp.2021.140

https://doi.org/10.1371/journal.pone.0280144

https://doi.org/10.1080/13623699.2021.1950519

https://www.thelancet.com/journals/lanpsy/article/PIIS2215-0366(24)00354-7/abstract

Line 16, 439,479

Line550

Line 604

Line571

Line665
Line661
