# Supplementary material for: Integrating psychosocial health into disaster risk management: Insights from COVID-19 in Durán, Ecuador
Source: PLoS One. 2026 Mar 27;21(3):e0343239. doi: 10.1371/journal.pone.0343239 (PMC13029789; doi:10.1371/journal.pone.0343239)
Supplement: Attachment — Submitted filename: Response to Reviewers_01292026.docx [file pone.0343239.s005.docx]

PONE-D-24-34167R1

Integrating Psychosocial Health into Disaster Risk Management: Insights from COVID-19 in Duran, Ecuador.

Below is our detailed response to the reviewer comments, with associated line numbers in the updated manuscript. We look forward to your review and response.

**Comments to the Author**

1. If the authors have adequately addressed your comments raised in a previous round of review and you feel that this manuscript is now acceptable for publication, you may indicate that here to bypass the “Comments to the Author” section, enter your conflict of interest statement in the “Confidential to Editor” section, and submit your "Accept" recommendation.

Reviewer #4: All comments have been addressed

Reviewer #5: (No Response)

Reviewer #6: (No Response)

2. Is the manuscript technically sound, and do the data support the conclusions?

The manuscript must describe a technically sound piece of scientific research with data that supports the conclusions. Experiments must have been conducted rigorously, with appropriate controls, replication, and sample sizes. The conclusions must be drawn appropriately based on the data presented.

Reviewer #4: Yes

Reviewer #5: Partly

Reviewer #6: Partly

3. Has the statistical analysis been performed appropriately and rigorously?

Reviewer #4: Yes

Reviewer #5: Yes

Reviewer #6: Yes

4. Have the authors made all data underlying the findings in their manuscript fully available?

The [PLOS Data policy](http://track.editorialmanager.com/CL0/http:%2F%2Fwww.plosone.org%2Fstatic%2Fpolicies.action%23sharing/1/010f019afcc74ec6-313853e6-0b31-49e1-9d56-436a569950c5-000000/BzB93q3EQBsvChUTCm7cstETyed6pZXr52yauo88BDE=240) requires authors to make all data underlying the findings described in their manuscript fully available without restriction, with rare exception (please refer to the Data Availability Statement in the manuscript PDF file). The data should be provided as part of the manuscript or its supporting information, or deposited to a public repository. For example, in addition to summary statistics, the data points behind means, medians and variance measures should be available. If there are restrictions on publicly sharing data—e.g. participant privacy or use of data from a third party—those must be specified.

Reviewer #4: Yes

Reviewer #5: Yes

Reviewer #6: Yes

5. Is the manuscript presented in an intelligible fashion and written in standard English?

PLOS ONE does not copyedit accepted manuscripts, so the language in submitted articles must be clear, correct, and unambiguous. Any typographical or grammatical errors should be corrected at revision, so please note any specific errors here.

Reviewer #4: Yes

Reviewer #5: Yes

Reviewer #6: Yes

6. Review Comments to the Author

Please use the space provided to explain your answers to the questions above. You may also include additional comments for the author, including concerns about dual publication, research ethics, or publication ethics. (Please upload your review as an attachment if it exceeds 20,000 characters)

Reviewer #4: (No Response)

Reviewer #5: Dear Author
Thank you for your submission. I read your manuscript and dropped some comments as following:

Introduction

The introduction has been substantially revised through the peer review process and now effectively establishes the research problem. It clearly articulates the gap between current DRM practices and psychosocial health integration, particularly in Global South contexts. However, I think there are some issues that need further attention, as follows:

- Some remaining redundancy (psychosocial health concept explained multiple times)

Thank you for this point. We removed lines 137-143 to avoid redundancy.

- Connection between climate hazards and COVID-19 could be more explicit

We added a paragraph including specific effects of compound climatic and pandemic risks globally and in our Latin American region.

Lines 61-65: “In line with the Sendai Framework for disaster risk reduction (DRR), humanitarian emergencies, climate and health crises, and compound risks offer critical opportunities to advance resilience in urban and health systems by addressing structural vulnerabilities, enhancing interinstitutional coordination, and integrating mental and psychosocial health into disaster risk reduction and management strategies.”

We also added updated references: Ford et al., 2021, Hartginger et al, 2023; Zavaleta et al., 2021, Uche Ezeh et al., 2021, Borg et al., 2021 and Palmeiro-Silva et al, 2022 on lines 94, 99 111, 104, 113, 491

- Limited discussion of why Durán specifically is relevant beyond prior climate studies

We would like to remark that the city of Durán has functioned as an urban laboratory, since 2018, for examining the intersection of climate hazards, social vulnerability, and COVID-19 through sustained community-based participatory research conducted by our team. This long-term engagement with the local government and communities has enabled a better understanding of the complex interactions among compound urban risks and contributes original empirical evidence to a field where research on intermediate cities in Latin America remains limited.

Line 132-136:“These efforts position the city as an urban laboratory for examining the complex interactions among climate risks, pandemics and social vulnerability in intermediate Latin America cities. Considering the paucity of original research and context-specific evidence, this study offers insights into how vulnerability factors interact during crises, informing strategies to strengthen urban resilience [29,34].”

- Some references dated (e.g., reference to 2010 census data for Durán population)

Line 158: The population of Duran has been updated to 303,910 residents, using data from the 2022 Ecuador’s Census.

- Could include more recent DRM integration literature post-2020 ( also in discussion)

This work also incorporates recent disaster risk management (DRM) and climate adaptation literature that integrates climate change with physical and mental health perspectives, strengthening the analysis of systemic and compounding risks in urban contexts. We also added references as UNDRR&WHO, 2023 on line 72 and Gray et al., 2021 on line 104.

- Missing some recent systematic reviews on pandemic mental health (also in discussion)

We have added several reviews on mental health:

Line 89: Zhang et al, 2022

Line 547: Duden et al, 2022

Line 481: Jia et al, 2024

Lines 104, 523: Gray et al, 2021

Lines 80, 99, 104, 109, 111: Lawrance et al, 2022

Methodology
Methodology is clear and reproducible, though the non-probabilistic sampling approach warrants careful consideration for interpreting results. There are some comments in this section to help for stronger methods as following:

- Please describe sample size calculation with justification

Sample size:

We thank the reviewer for this comment. The sample size was determined based on the logistic and operational capacity of the field campaigns, consistent with the non-probabilistic convenience sampling strategy employed. In this context, the sample size was not based on a formal a priori calculation, as is typical in probabilistic study designs. Nevertheless, with the achieved sample size (n = 340), the study had adequate statistical power (>80%) to detect associations of moderate magnitude (odds ratios approximately between 1.5 and 2.0) at a 5% significance level for the main dichotomous outcomes, contributing to the stability of the estimated logistic regression models. The “Strengths and Limitations” section notes that associations of smaller magnitude were estimated with wide confidence intervals and non-significant results, primarily due to the low number of events in some categories, which is expected given the sample size and data distribution.

The sample size was determined based on the logistic and operational capacity of the field campaigns, consistent with the non-probabilistic convenience sampling strategy employed.

Line 182-185:“Thus, sample size was determined based on the operational capacity of the field campaigns, consistent with the non-probabilistic convenience sampling strategy employed. See Figure 1 for description of the study site and sampling locations [59].”

Lines 581-600, in the "Strengths and Limitations” section notes:

Finally, although the sample size obtained provided adequate statistical power to detect associations of moderate magnitude, some associations, particularly those involving less frequent categories, were estimated with wide confidence intervals and non-significant results. This reflects limited precision due to data scarcity rather than the absence of an underlying association, which has been considered in the interpretation of findings.

- Limited discussion of how multicollinearity among vulnerability variables was addressed

Thank you for the questions to help clarify the data processing. Before conducting multivariable analyses, we assessed multicollinearity among independent variables representing individual vulnerability, urban sensitivity, and adaptive capacity. We examined correlations between covariates and the outcomes (stress and depression), as well as inter-covariate associations, using measures appropriate to variable scale (Spearman or Kendall coefficients for ordinal variables and Cramér’s V for nominal variables). Most associations were weak (<0.2), with the exception of sex and occupational status, which showed a moderate association (~0.4). Although individual correlation values are not reported, this information has now been explicitly clarified in the Correlation Analysis section.

We include in the manuscript:

Line 287: “as well as to evaluate potential multicollinearity among vulnerability-related covariates…”

-There is no mention of data validation procedures

The steps described, data validation and quality control procedures were applied prior to analysis. These included verification of data completeness, detection of out-of-range values, and consistency checks between related variables (e.g., demographic and clinical information). Duplicate records were identified and removed using unique participant codes, and the final dataset was anonymized before analysis. These procedures have now been explicitly described and clarified in the Methods section to improve transparency and reproducibility.

On lines 311-314, we have added a concise description of the data validation procedure.

“Prior to analysis, data validation procedures were conducted,including checks for completeness and internal consistency, with duplicate records removed using unique participant identifiers.”

In lines 311-314, we explain the data preprocessing:

“All statistical analyses were conducted using R (version 4.4.2). Data were validated prior to analysis, and assumptions for each statistical test, including variable scale and distribution, were assessed. When assumptions of normality or homoscedasticity were not met, appropriate non-parametric methods were applied. Missing data were not imputed; analyses were restricted to complete cases.”

Results

Results are presented accurately and clearly with appropriate use of tables and figures. Data presentation is generally well-organized. There are just some comments to help you for better result presentation as following:

- Tables 3 & 4 are dense; some extreme OR values (>1000) indicate complete separation in logistic regression, limiting interpretability.

Tables 3 and 4 were revised to improve interpretability by retaining only stable and interpretable odds ratio estimates. Results affected by complete or quasi-complete separation, or presenting extreme confidence intervals, were not reported as odds ratios and this clarification has been explicitly included in the Results section.

Line 380: Table 3 presents the regression model predicting moderate stress; estimates for high stress were not reported due to very low event counts (<10) and limited interpretability.

Line 380: The new Table 3 is included in the manuscript.

Line 408: New table 4 is included in the manuscript.

- Some confidence intervals extremely wide, suggesting sparse data in certain subgroups.

The presence of very wide confidence intervals has been explicitly indicated in the “Strengths and Limitations” section starting on line 581, clarifying that these estimates are primarily driven by sparse data and low event counts in certain subgroups rather than by the absence of an underlying association.

Discussion

The discussion is comprehensive, well-structured, and effectively interprets findings in context of existing literature. Recent revisions have significantly improved regional contextualization. There are more issues to consider:

- Limited discussion of potential reverse causality (does depression predict lower vaccination rates or vice versa?)

Line 482-488: Thank you for this relevant question. We have revised the manuscript to include recent global literature showing mixed findings, with several studies reporting lower COVID-19 vaccination uptake and higher hesitancy among individuals with depression and anxiety. We also incorporated recent evidence from Durán (Orellana-Manzano et al., 2026), which reported high willingness to follow vaccination guidance, reflecting strong trust in local authorities and health providers despite limited knowledge of COVID-19 transmission. Finally, we clarify that the present study is designed to examine associations rather than to establish causal relationships.

- Could discuss intersectionality more explicitly (overlapping gender, age, economic vulnerabilities)

Lines 512-518: Thank you for your comment. Intersectionality is a critical concept in disaster risk reduction, public health, and climate change adaptation in urban contexts. We have added a paragraph highlighting the study contribution to intersectionality research by identifying how multiple, intertwined vulnerability factors shape mental health outcomes, while also acknowledging that power dynamics, violence, and safety conditions are critical determinants that must be considered in the design and implementation of interventions.

“Duran’s study contributes to intersectionality research by moving beyond the individual level to examine how structural and environmental inequities at the urban scale shape vulnerability [112,113]. Showing that gender, age, health status, inadequate urban infrastructure, residence in informal neighborhoods, and access to knowledge and health information interact to produce distinct patterns of vulnerability that help explain mental health disparities during health-related crises. Besides urban power asymmetries, violence, and the availability of safe spaces emerge as critical contextual factors for the design and implementation of effective interventions [114].”

- Implications for disaster preparedness could be more concrete

To elaborate on implications for disaster preparedness, we added lines 711-715 to the following sentence:

Line 710: “Strengthening community social networks at community level, simultaneously builds resilience to both health emergencies and climate hazards [117].”

Line 711-715: “The Entre Nosotras initiative illustrates culturally adapted, scalable strategies for Ecuador and other Latin American cities by shifting from individual to group-based interventions aligned with community preferences, incorporating shared childcare arrangements, scheduling activities during evenings and weekends, and strengthening safe community spaces to support psychosocial wellbeing [118].”

- Limited discussion of cultural adaptations needed for interventions in Durán context

We added specific interventions at the family and community level that we believe are needed in our cultural context.

Lines 535-542: “The strategies may include psychosocial first aid, skill‑building workshops, family communication plans, and preparedness kits (including children’s items), to foster safety, support, and resilience before disasters occur. Strengthening community social networks at community level, simultaneously builds resilience to both health emergencies and climate hazards [117]. The Entre Nosotras initiative illustrates culturally adapted, scalable strategies for Ecuador and other Latin American cities by shifting from individual to group-based interventions aligned with community preferences, incorporating shared childcare arrangements, scheduling activities during evenings and weekends, and strengthening safe community spaces to support psychosocial wellbeing [118].”

Conclusions
Well written. Conclusion is evidence-based and consistent with research objectives, appropriately synthesizing findings and providing actionable recommendations.

References and Citations
References are properly formatted and citations are comprehensive, though formatting consistency has been improved.

- Some older citations (2010 census data for Durán) could be updated with more recent statistics if available.

Line 158: We have updated the population of Duran to 303,910 according to data from the 2022 Census

There are more comments:
- Missing some recent COVID-19 mental health systematic reviews (2021-2024)

Lines 80, 99, 104, 109, 111: We added a narrative review (Lawrance et al., 2022) of the impacts of climate change and COVID-19 on mental health and emotional wellbeing, presenting current evidence, and its implications.

Line 547: We added a systematic review Global impact of the COVID-19 pandemic on mental health services (Duden et al, 2022)

Line 89: We added a systematic review on Mental disorder symptoms during the COVID-19 pandemic in Latin America (Zhang et al, 2022)

- Limited representation of Global South-specific disaster resilience literature

Lines 111, 491: We have added the scoping review of Palmeiro Silva et al. (2022) that found that climate change mental health impacts in South America are associated with trauma, depression, and anxiety, however, evidence is limited and lacking transdisciplinarity.

Line 99, 104, 111, 113: We have included the scoping review by Borg et al. (2021), which examines the health impacts of climate-related hazards (floods. heat waves, extreme events) in informal settlements in low- and middle-income countries, highlighting major gaps in evidence on health adaptation and risk-reduction interventions.

-Reviewer #6: Line 154: The study was conducted in 2021; therefore, some of the findings and a portion of the referenced literature may now be considered outdated. Updating these elements could strengthen the manuscript.

We have added new references published in the last year to corroborate our existing writing.

Line 89: de Sousa et al, 2025

Line 130: Cepella et al, 2025

Line 423: Howard et al, 2025

Line 441: Sanders et al, 2025

Line 513: White et al, 2025

Line 516: Buenaño et al, 2025

-Line 154: I continue to have reservations regarding the representativeness of the non-probabilistic convenience sampling approach used for the quantitative survey. Clarification or justification of this method would be helpful.

The sample size was determined based on the logistic and operational capacity of the field campaigns, consistent with the non-probabilistic convenience sampling strategy employed. In this context, the sample size was not based on a formal a priori calculation, as is typical in probabilistic study designs. Nevertheless, with the achieved sample size (n = 340), the study had adequate statistical power (>80%) to detect associations of moderate magnitude (odds ratios approximately between 1.5 and 2.0) at a 5% significance level for the main dichotomous outcomes, contributing to the stability of the estimated logistic regression models.

-Line 164: The rationale for selecting community centers as recruitment sites appears somewhat subjective. Further explanation on how logistical feasibility, infrastructure adequacy, and safety considerations informed this decision would enhance clarity.

The field campaign (Figure 2) required an open space with sufficient capacity to install tents and conduct all study phases, including informed consent, app installation, survey administration, and blood sample collection; therefore, sites with adequate facilities and safety conditions for participants and the research team were selected.

-Line 168: It is unclear why 106 out of the 446 individuals who initially participated did not complete the study and were subsequently excluded. Providing additional detail on the reasons for non-completion would strengthen the methodology section.

Line 187-191: Of the 446 individuals initially enrolled, 106 participants were excluded due to incomplete survey data. Data quality control indicated that non-completion was primarily due to participants choosing not to continue or answer all questions. The final analytical sample comprised 340 participants who completed all required instruments and met the inclusion criterion of being 12 years of age or older.

-Line 176: The explanation that sites were selected to capture urban neighborhood diversity and based on logistical feasibility may still appear subjective. Additional justification on how these criteria were applied would be beneficial.

To minimize subjectivity, site selections followed a predefined criteria applied across locations. Neighborhoods were stratified to represent diverse urban infrastructure (urban typology) and vulnerability contexts from previous studies (Borbor-Cordova et.al, 2020). Ensuring representation of consolidated, growing, dense, and informal urban contexts. In each stratum, sites were selected based on logistic feasibility and safety conditions for participants and research team.

-Line 183: Please clarify the type of consent obtained and how it was documented electronically to ensure transparency and adherence to ethical standards.

Informed verbal consent was obtained from all participants prior to data collection. Consent was documented electronically within the survey platform, where interviewers recorded consent before proceeding with the questionnaire, in accordance with the approved ethical protocol. This has been updated in the manuscript.
